# The genome sequence of the Oak-tree Pug, *Eupithecia dodoneata* (Guenée, 1858)

**DOI:** 10.12688/wellcomeopenres.19245.1

**Published:** 2023-03-23

**Authors:** Douglas Boyes, Peter W.H. Holland, University of Oxford and Wytham Woods Genome Acquisition Lab, Darwin Tree of Life Barcoding collective, Wellcome Sanger Institute Tree of Life programme, Wellcome Sanger Institute Scientific Operations: DNA Pipelines collective, Tree of Life Core Informatics collective, Darwin Tree of Life Consortium

**Affiliations:** 1 UK Centre for Ecology & Hydrology, Wallingford, England, UK; 2 University of Oxford, Oxford, England, UK

**Keywords:** Eupithecia dodoneata, Oak-tree Pug, genome sequence, chromosomal, Lepidoptera

## Abstract

We present a genome assembly from an individual male *Eupithecia dodoneata* (the Oak-tree Pug; Arthropoda; Insecta; Lepidoptera; Geometridae). The genome sequence is 353.7 megabases in span. Most of the assembly is scaffolded into 31 chromosomal pseudomolecules, including the assembled Z sex chromosome. The mitochondrial genome has also been assembled and is 15.3 kilobases in length.

## Species taxonomy

Eukaryota; Metazoa; Ecdysozoa; Arthropoda; Hexapoda; Insecta; Pterygota; Neoptera; Endopterygota; Lepidoptera; Glossata; Ditrysia; Geometroidea; Geometridae; Larentiinae; *Eupithecia*; *Eupithecia dodoneata* (Guenée, 1858) (NCBI:txid934845).

## Background

The Oak-tree Pug *Eupithecia dodoneata* is a small and delicately-patterned moth in the family Geometridae found widely across Europe, with scattered records from Asia Minor and North Africa ( GBIF Secretariat, 2022). The forewings have a light grey ground colour, crossed by bands of brown and dark grey, with a prominent black discal spot. In the UK, the moth is common is woodlands and suburban areas in the south of England and Wales where oaks are present ( Riley & Prior, 2003). The moth is univoltine in the UK, with adults on the wing in May and June, larvae developing through summer, and pupae overwintering. The commonest larval food plants in the north of Europe are pedunculate oak *Quercus robur* and holm oak *Q. ilex*, with indications from rearing that larvae also eat the fleshy sepals around the fruits of hawthorn *Crataegus monogyna* ( Haggett, 1992). In Turkey, downy oak *Q. pubescens* is also used as a food plant ( Torun & Seven Çalişkan, 2016) and in Italy *E. dodoneata* was found to be the commonest oak-feeding species in woodlands of cork oak *Q. suber* ( Scalercio, 2022).

A genome sequence for *E. dodoneata* will facilitate studies investigating molecular adaptations to oak feeding and will contribute to the growing set of genomic resources for Lepidoptera.

## Genome sequence report

**Figure 1.**
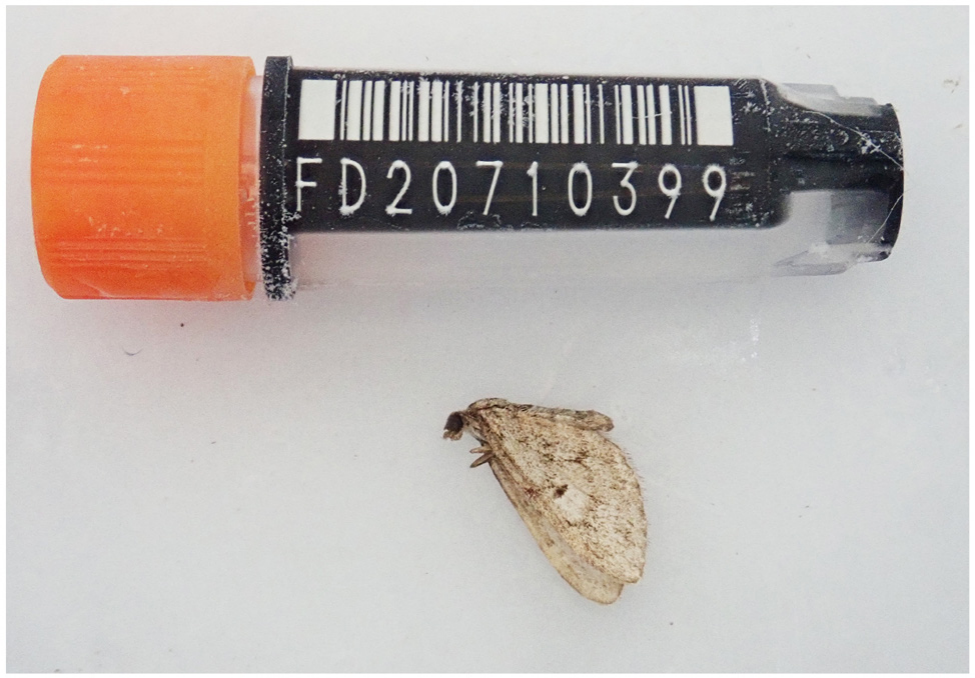
Photograph of the *Eupithecia dodoneata* (ilEupDodo1) specimen used for genome sequencing.

The genome was sequenced from one male *Eupithecia dodoneata* ( Figure 1) collected from Wytham Woods, Oxfordshire, UK (latitude 51.77, longitude –1.32). A total of 53-fold coverage in Pacific Biosciences single-molecule HiFi long reads was generated. Primary assembly contigs were scaffolded with chromosome conformation Hi-C data. Manual assembly curation corrected five missing or mis-joins, reducing the scaffold number by 5.71%.

**Figure 2.**
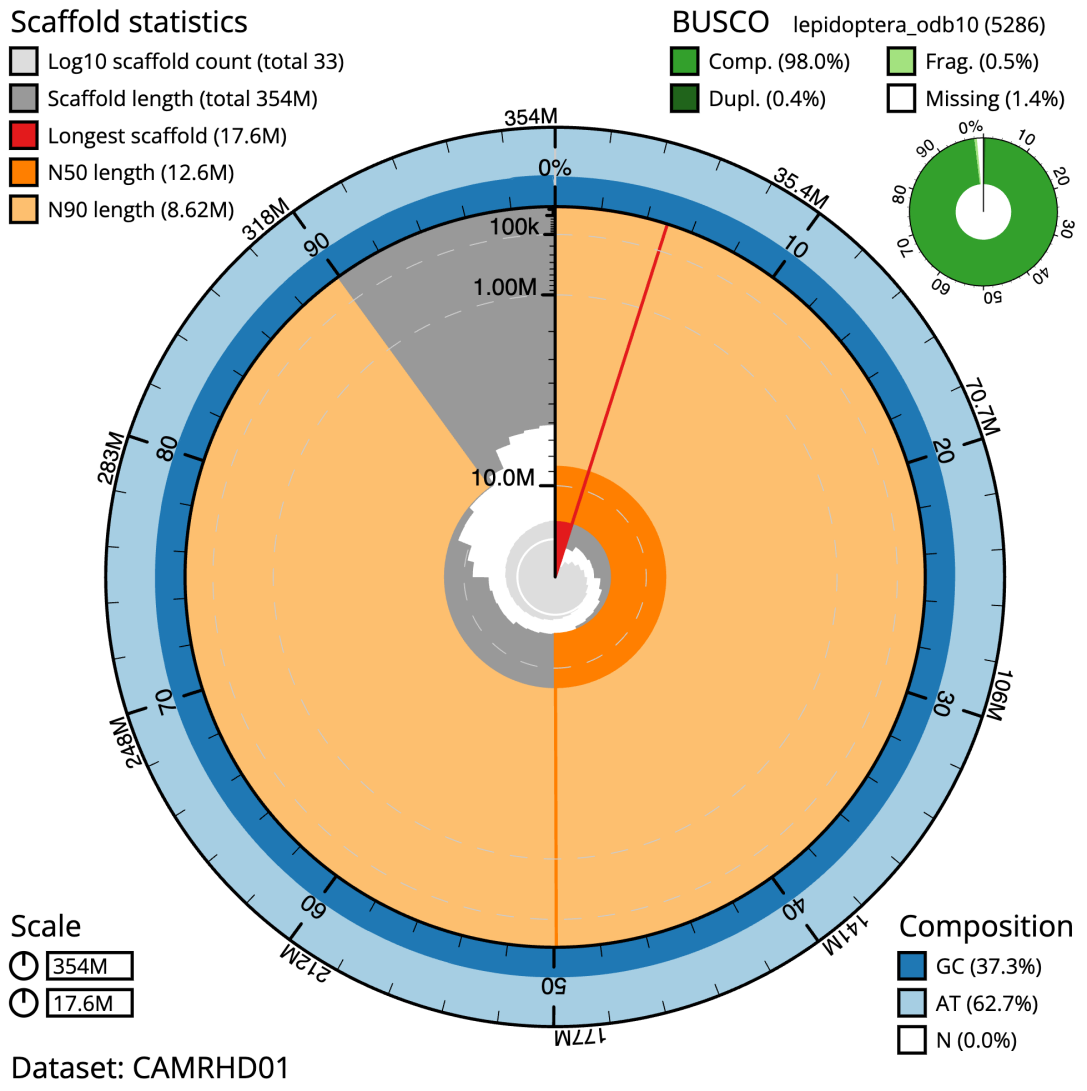
Genome assembly of *Eupithecia dodoneata*, ilEupDodo1.1: metrics. The BlobToolKit Snailplot shows N50 metrics and BUSCO gene completeness. The main plot is divided into 1,000 size-ordered bins around the circumference with each bin representing 0.1% of the 353,656,369 bp assembly. The distribution of scaffold lengths is shown in dark grey with the plot radius scaled to the longest scaffold present in the assembly (17,599,884 bp, shown in red). Orange and pale-orange arcs show the N50 and N90 scaffold lengths (12,556,972 and 8,615,116 bp), respectively. The pale grey spiral shows the cumulative scaffold count on a log scale with white scale lines showing successive orders of magnitude. The blue and pale-blue area around the outside of the plot shows the distribution of GC, AT and N percentages in the same bins as the inner plot. A summary of complete, fragmented, duplicated and missing BUSCO genes in the lepidoptera_odb10 set is shown in the top right. An interactive version of this figure is available at https://blobtoolkit.genomehubs.org/view/ilEupDodo1.1/dataset/CAMRHD01/snail.

The final assembly has a total length of 353.7 Mb in 33 sequence scaffolds with a scaffold N50 of 12.6 Mb ( Table 1). Most (99.98%) of the assembly sequence was assigned to 31 chromosomal-level scaffolds, representing 30 autosomes, and the Z sex chromosome. Chromosome-scale scaffolds confirmed by the Hi-C data are named in order of size ( Figure 2– Figure 5; Table 2). While not fully phased, the assembly deposited is of one haplotype. Contigs corresponding to the second haplotype have also been deposited. The estimated Quality Value (QV) of the final assembly is 70.8 with *k*-mer completeness of 100%, and the assembly has a BUSCO v5.3.2 ( Manni *et al.*, 2021) completeness of 98.0% (single 97.6%, duplicated 0.4%) using the lepidoptera_odb10 reference set ( *n* = 5,286).

**Table 1.** Genome data for *Eupithecia dodoneata*, ilEupDodo1.1.

Project accession data		
Assembly identifier	ilEupDodo1.1	
Species	*Eupithecia dodoneata*	
Specimen	ilEupDodo1	
NCBI taxonomy ID	934845	
BioProject	PRJEB55027	
BioSample ID	SAMEA10979161	
Isolate information	ilEupDodo1 (DNA sequencing and Hi-C scaffolding)	
Assembly metrics *		*Benchmark*
Consensus quality (QV)	70.8	*≥ 50*
*k*-mer completeness	100%	*≥ 95%*
BUSCO **	C:98.0%[S:97.6%,D:0.4%], F:0.5%,M:1.4%,n:5,286	*C ≥ 95%*
Percentage of assembly mapped to chromosomes	99.98%	*≥ 95%*
Sex chromosomes	Z chromosome	*localised homologous pairs*
Organelles	Mitochondrial genome assembled	*complete single alleles*
**Raw data accessions**		
PacificBiosciences SEQUEL II	ERR10008906	
Hi-C Illumina	ERR10015063	
**Genome assembly**		
Assembly accession	GCA_947044415.1	
*Accession of alternate haplotype*	GCA_947044255.1	
Span (Mb)	353.7	
Number of contigs	40	
Contig N50 length (Mb)	12.6	
Number of scaffolds	33	
Scaffold N50 length (Mb)	12.6	
Longest scaffold (Mb)	17.6	

* Assembly metric benchmarks are adapted from column VGP-2020 of “Table 1: Proposed standards and metrics for defining genome assembly quality” from ( Rhie *et al.*, 2021). ** BUSCO scores based on the lepidoptera_odb10 BUSCO set using v5.3.2. C = complete [S = single copy, D = duplicated], F = fragmented, M = missing, n = number of orthologues in comparison. A full set of BUSCO scores is available at https://blobtoolkit.genomehubs.org/view/ilEupDodo1.1/dataset/CAMRHD01/busco.

**Figure 3.**
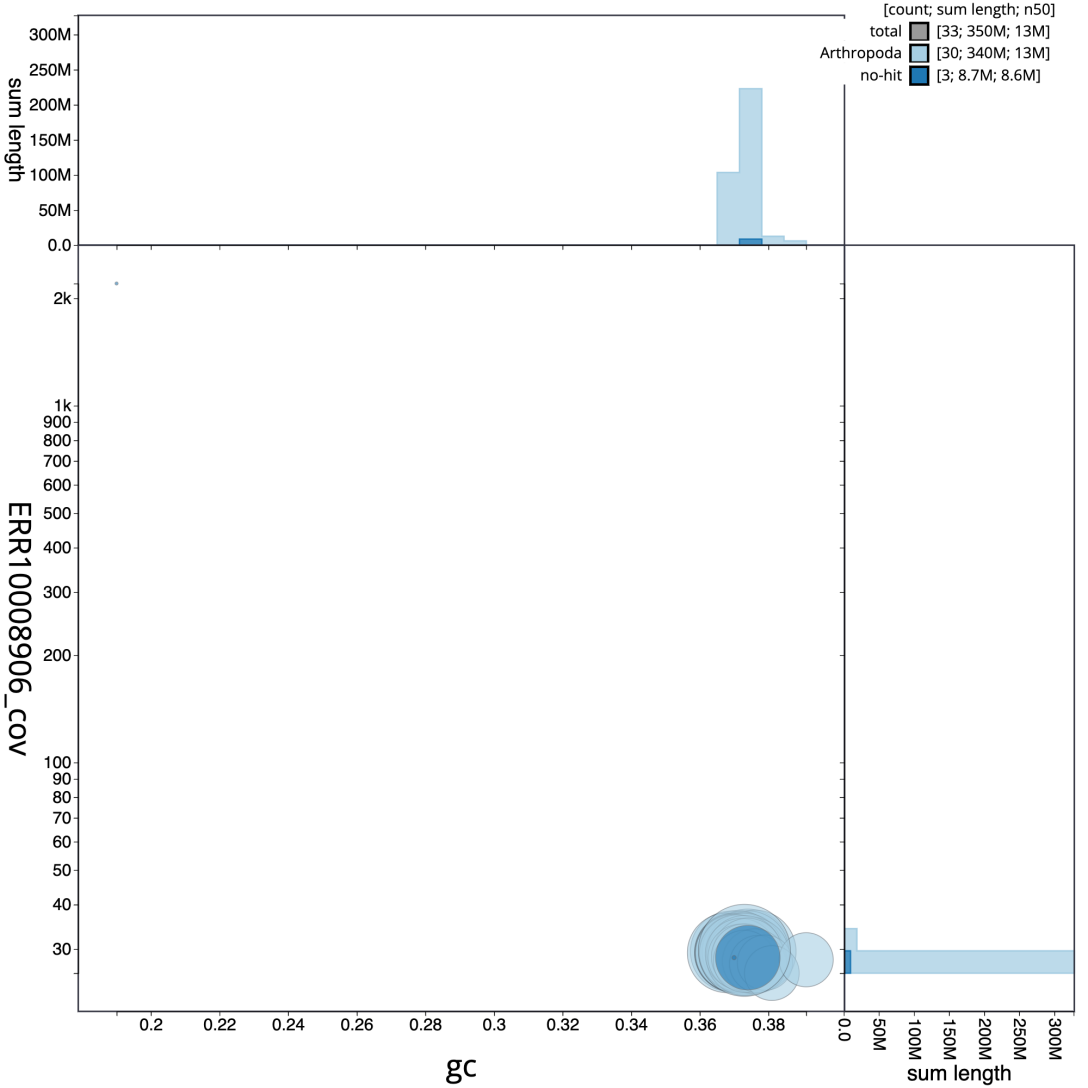
Genome assembly of *Eupithecia dodoneata*, ilEupDodo1.1: GC coverage. BlobToolKit GC-coverage plot. Scaffolds are coloured by phylum. Circles are sized in proportion to scaffold length. Histograms show the distribution of scaffold length sum along each axis. An interactive version of this figure is available at https://blobtoolkit.genomehubs.org/view/ilEupDodo1.1/dataset/CAMRHD01/blob.

**Figure 4.**
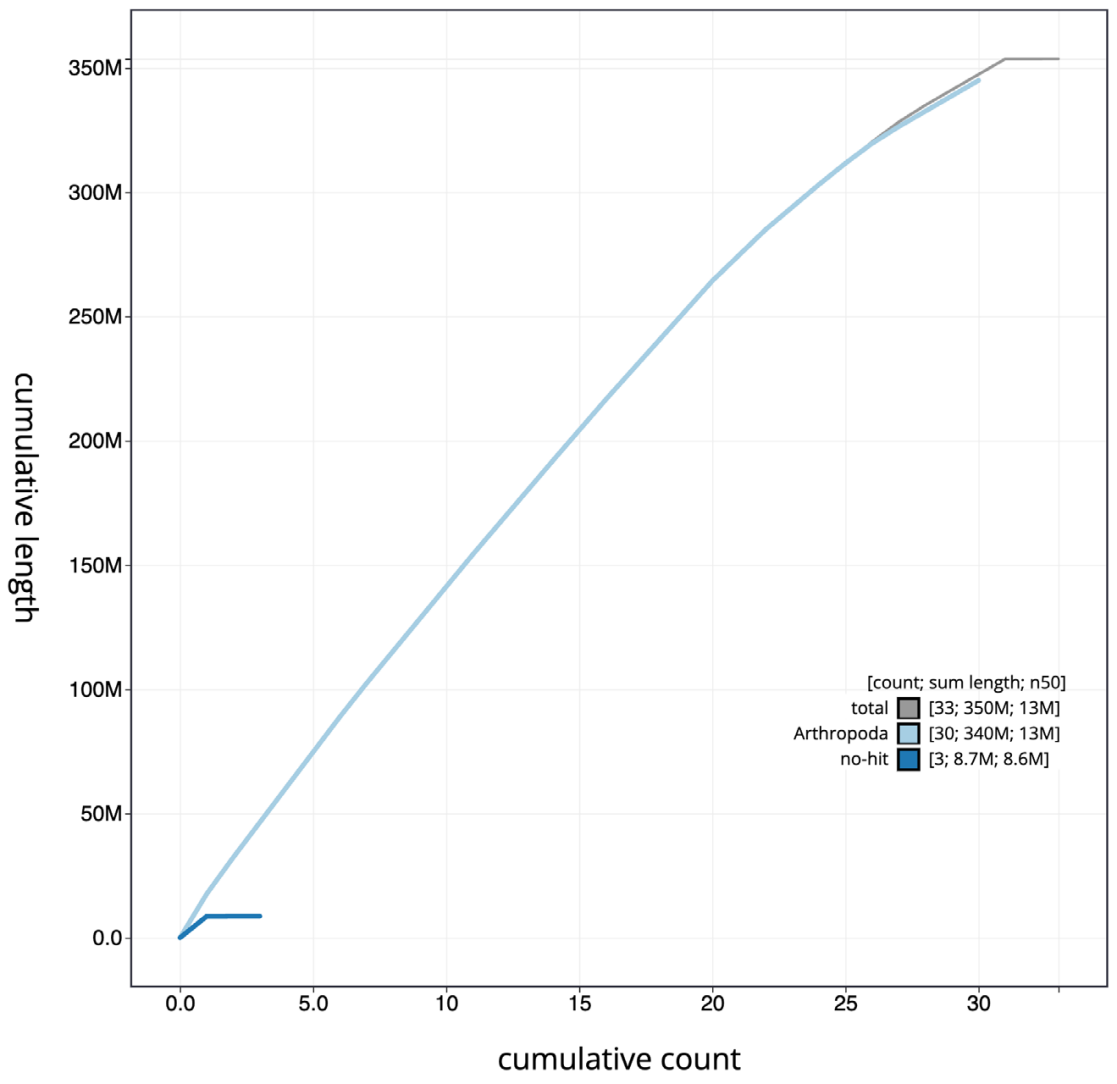
Genome assembly of *Eupithecia dodoneata*, ilEupDodo1.1: cumulative sequence. BlobToolKit cumulative sequence plot. The grey line shows cumulative length for all scaffolds. Coloured lines show cumulative lengths of scaffolds assigned to each phylum using the buscogenes taxrule. An interactive version of this figure is available at https://blobtoolkit.genomehubs.org/view/ilEupDodo1.1/dataset/CAMRHD01/cumulative.

**Figure 5.**
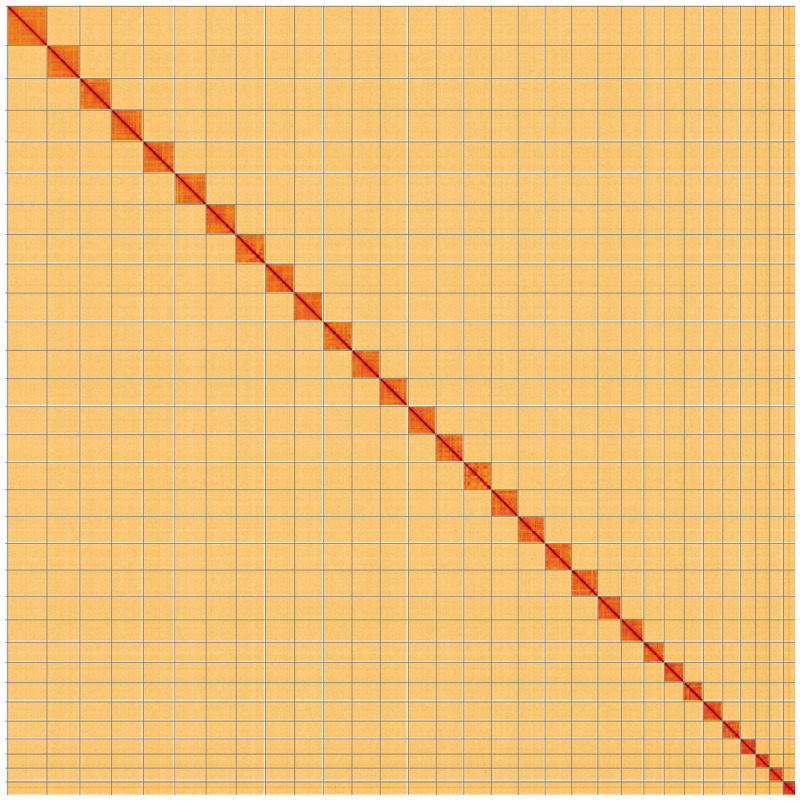
Genome assembly of *Eupithecia dodoneata*, ilEupDodo1.1: Hi-C contact map. Hi-C contact map of the ilEupDodo1.1 assembly, visualised using HiGlass. Chromosomes are shown in order of size from left to right and top to bottom. An interactive version of this figure may be viewed at https://genome-note-higlass.tol.sanger.ac.uk/l/?d=RGycXTw0Tf-PT5rzPINZKA.

**Table 2.** Chromosomal pseudomolecules in the genome assembly of *Eupithecia dodoneata*, ilEupDodo1.

INSDC accession	Chromosome	Size (Mb)	GC%
OX345656.1	1	14.72	37.4
OX345657.1	2	14.22	37.3
OX345658.1	3	14.22	37.6
OX345659.1	4	14.06	37.4
OX345660.1	5	14.01	37.1
OX345661.1	6	13.47	36.9
OX345662.1	7	13.2	36.8
OX345663.1	8	13	37
OX345664.1	9	12.92	37.2
OX345665.1	10	12.81	37
OX345666.1	11	12.6	37.1
OX345667.1	12	12.56	37.2
OX345668.1	13	12.51	37.3
OX345669.1	14	12.41	37
OX345670.1	15	12.32	37.3
OX345671.1	16	12.03	37.1
OX345672.1	17	12.01	37.3
OX345673.1	18	11.93	37.5
OX345674.1	19	11.76	37.3
OX345675.1	20	10.56	37.2
OX345676.1	21	10.07	37.4
OX345677.1	22	9.05	37.2
OX345678.1	23	9.02	37.3
OX345679.1	24	8.62	37.4
OX345680.1	25	8.62	37.6
OX345681.1	26	7.98	37.3
OX345682.1	27	6.75	37.7
OX345683.1	28	6.33	37.9
OX345684.1	29	6.2	38.1
OX345685.1	30	6.07	39.1
OX345655.1	Z	17.6	37.3
OX345686.1	MT	0.02	19

## Methods

### Sample acquisition and nucleic acid extraction

A male *Eupithecia dodoneata* (ilEupDodo1) was collected from Wytham Woods, Oxfordshire, UK (biological vice-county: Berkshire) (latitude 51.77, longitude –1.32) on 28 May 2021. The specimen was taken from woodland habitat by Douglas Boyes (University of Oxford) using a light trap. The specimen was identified by the collector and snap-frozen on dry ice.

DNA was extracted at the Tree of Life laboratory, Wellcome Sanger Institute (WSI). The ilEupDodo1 sample was weighed and dissected on dry ice with tissue set aside for Hi-C sequencing. Whole organism tissue was disrupted using a Nippi Powermasher fitted with a BioMasher pestle. High molecular weight (HMW) DNA was extracted using the Qiagen MagAttract HMW DNA extraction kit. HMW DNA was sheared into an average fragment size of 12–20 kb in a Megaruptor 3 system with speed setting 30. Sheared DNA was purified by solid-phase reversible immobilisation using AMPure PB beads with a 1.8X ratio of beads to sample to remove the shorter fragments and concentrate the DNA sample. The concentration of the sheared and purified DNA was assessed using a Nanodrop spectrophotometer and Qubit Fluorometer and Qubit dsDNA High Sensitivity Assay kit. Fragment size distribution was evaluated by running the sample on the FemtoPulse system.

### Sequencing

Pacific Biosciences HiFi circular consensus DNA sequencing libraries were constructed according to the manufacturers’ instructions. DNA sequencing was performed by the Scientific Operations core at the WSI on Pacific Biosciences SEQUEL II (HiFi) instrument. Hi-C data were also generated from tissue of ilEupDodo1 using the Arima v2 kit and sequenced on the Illumina NovaSeq 6000 instrument.

### Genome assembly, curation and evaluation

Assembly was carried out with Hifiasm ( Cheng *et al.*, 2021) and haplotypic duplication was identified and removed with purge_dups ( Guan *et al.*, 2020). The assembly was scaffolded with Hi-C data ( Rao *et al.*, 2014) using YaHS ( Zhou *et al.*, 2023). The assembly was checked for contamination as described previously ( Howe *et al.*, 2021). Manual curation was performed using HiGlass ( Kerpedjiev *et al.*, 2018) and Pretext ( Harry, 2022). The mitochondrial genome was assembled using MitoHiFi ( Uliano-Silva *et al.*, 2022), which performed annotation using MitoFinder ( Allio *et al.*, 2020). To evaluate the assembly, MerquryFK was used to estimate consensus quality (QV) scores and *k*-mer completeness ( Rhie *et al.*, 2020). The genome was analysed and BUSCO scores ( Simão *et al.*, 2015; Manni *et al.*, 2021) were generated within the BlobToolKit environment ( Challis *et al.*, 2020). Table 3 contains a list of software tool versions and sources.

**Table 3.** Software tools: versions and sources.

Software tool	Version	Source
BlobToolKit	4.0.7	https://github.com/blobtoolkit/ blobtoolkit
BUSCO	5.3.2	https://gitlab.com/ezlab/busco
Hifiasm	0.16.1-r375	https://github.com/chhylp123/ hifiasm
HiGlass	1.11.6	https://github.com/higlass/higlass
Merqury	MerquryFK	https://github.com/thegenemyers/ MERQURY.FK
MitoHiFi	2	https://github.com/marcelauliano/ MitoHiFi
PretextView	0.2	https://github.com/wtsi-hpag/ PretextView
purge_dups	1.2.3	https://github.com/dfguan/purge_ dups
YaHS	yahs- 1.1.91eebc2	https://github.com/c-zhou/yahs

### Ethics and compliance issues

The materials that have contributed to this genome note have been supplied by a Darwin Tree of Life Partner. The submission of materials by a Darwin Tree of Life Partner is subject to the Darwin Tree of Life Project Sampling Code of Practice. By agreeing with and signing up to the Sampling Code of Practice, the Darwin Tree of Life Partner agrees they will meet the legal and ethical requirements and standards set out within this document in respect of all samples acquired for, and supplied to, the Darwin Tree of Life Project. All efforts are undertaken to minimise the suffering of animals used for sequencing. Each transfer of samples is further undertaken according to a Research Collaboration Agreement or Material Transfer Agreement entered into by the Darwin Tree of Life Partner, Genome Research Limited (operating as the Wellcome Sanger Institute), and in some circumstances other Darwin Tree of Life collaborators.

## Data Availability

European Nucleotide Archive: *Eupithecia dodoneata* (oak tree pug). Accession number PRJEB55027; https://identifiers.org/ena.embl/PRJEB55027. ( Wellcome Sanger Institute, 2022) The genome sequence is released openly for reuse. The *Eupithecia dodoneata* genome sequencing initiative is part of the Darwin Tree of Life (DToL) project. All raw sequence data and the assembly have been deposited in INSDC databases. The genome will be annotated using available RNA-Seq data and presented through the Ensembl pipeline at the European Bioinformatics Institute. Raw data and assembly accession identifiers are reported in Table 1.

## Author information

Members of the University of Oxford and Wytham Woods Genome Acquisition Lab are listed here: https://doi.org/10.5281/zenodo.4789928.

Members of the Darwin Tree of Life Barcoding collective are listed here: https://doi.org/10.5281/zenodo.4893703.

Members of the Wellcome Sanger Institute Tree of Life programme are listed here: https://doi.org/10.5281/zenodo.4783585.

Members of Wellcome Sanger Institute Scientific Operations: DNA Pipelines collective are listed here: https://doi.org/10.5281/zenodo.4790455.

Members of the Tree of Life Core Informatics collective are listed here: https://doi.org/10.5281/zenodo.5013541.

Members of the Darwin Tree of Life Consortium are listed here: https://doi.org/10.5281/zenodo.4783558.
