# Supplementary material for: The genome sequence of the Oak-tree Pug, Eupithecia dodoneata (Guenée, 1858)
Source: Wellcome Open Res. 2023 Mar 23;8:133. [Version 1] doi: 10.12688/wellcomeopenres.19245.1 (PMC10352626; doi:10.12688/wellcomeopenres.19245.1)
Supplement: Peer review history

# Peer review: Reviewer response for version 1

**DOI:** 10.21956/wellcomeopenres.21328.r62924

**Published:** 2023-07-17

**Authors:** Kathy Darragh

**Affiliations:** 1 University of California Davis, Davis, California, USA

The authors present a genome assembly for *Eupithecia dodoneata*, a small geometrid moth with a broad geographic distribution. Previous studies have found that *E. dodoneata* feeds on different larval food plants, which varies geographically. The genome assembly will facilitate future studies of how these populations are adapted to feeding on different plant species.

 The pipeline used for genome assembly is standard and the protocol is well-described in the methods. The assembly produce is chromosomal and high quality. The authors provide a link to ENA where the assembly and other associated data is available.

 The authors mention that the annotation will be available in the future. I wonder if it would be possible to report the annotation in this same report when available to compare BUSCO scores to the genome.

Are sufficient details of methods and materials provided to allow replication by others?

Yes

Is the rationale for creating the dataset(s) clearly described?

Yes

Are the datasets clearly presented in a useable and accessible format?

Yes

Are the protocols appropriate and is the work technically sound?

Yes

Reviewer Expertise:

Evolutionary biology, chemical ecology, genetics, molecular biology

I confirm that I have read this submission and believe that I have an appropriate level of expertise to confirm that it is of an acceptable scientific standard.

# Peer review: Reviewer response for version 1

**DOI:** 10.21956/wellcomeopenres.21328.r55719

**Published:** 2023-04-11

**Authors:** Changhai Sun

**Affiliations:** 1 Laboratory of Insect Taxonomy & Aquatic Insects, College of Plant Protection, Nanjing Agricultural University, Nanjing, China

The paper presents the genome assembly of *Eupithecia dodoneata* and provides important information about its size and scaffolding.

 The methods for the sequencing and assembly of the species are appropriate, and the methods used in this paper are conventional ones, others can also use these methods to obtain similar results. Moreover, the authors also present the weblink to download the data concerning *Eupithecia dodoneata*.

 However, I would suggest that authors should describe the method of Z chromosome identification in the section of material and methods, and explain if the species has been surveyed in the genome and how the heterozygosity is.

Are sufficient details of methods and materials provided to allow replication by others?

Yes

Is the rationale for creating the dataset(s) clearly described?

Yes

Are the datasets clearly presented in a useable and accessible format?

Yes

Are the protocols appropriate and is the work technically sound?

Yes

Reviewer Expertise:

Insect systematics

I confirm that I have read this submission and believe that I have an appropriate level of expertise to confirm that it is of an acceptable scientific standard.
